# Robot-assisted repair for ureteroileal anastomosis stricture after cystectomy: technical points

**DOI:** 10.1590/S1677-5538.IBJU.2018.0794

**Published:** 2019-12-17

**Authors:** Juan Garisto, Riccardo Bertolo, Mohamed Eltemamy, Rebecca Campbell, Jihad Kaouk

**Affiliations:** 1 Department of Urology, Cleveland Clinic, Cleveland, Ohio, United States

## Abstract

**AIM:**

Uretero-ileal anastomosis strictures (UAS) occur in 3 to 11% of patients who undergo ileal conduit urinary diversion after cystectomy. We aimed to demonstrate our surgical technique for robotic repair of UAS after cystectomy, focusing on the technical points.

**MATERIALS AND METHODS:**

We present the case of a 75 year-old male with right hydronephrosis status post cystectomy with ileal conduit urinary diversion. Da Vinci Si® surgical system (Intuitive Surgical, Sunnyvale, CA) was docked and access into the abdominal cavity was gained. Uretero-ileal anastomosis was identified followed by ureteral stent visualization guiding the dissection. Stent was cut and further ureteral dissection was performed to maximize the length. Ureter was spatulated and specimen was sent for frozen section. Ileal conduit was incised at the site of the planned ureteral reimplantation. A new ureteral stent was inserted and the uretero-ileal anastomosis was performed. Thereafter, the previous site of the right ureteral anastomosis was closed.

**RESULTS:**

Operative time was 120 minutes. Blood loss was 60mL. No perioperative complications occurred. Patient was discharged on postoperative day 1. Technical points for outcomes optimization during UAS robotic repair: 1) Preoperative placement of a ureteral stent is required for guidance and urinary diversion, 2) Port placement should be tailored according to the previous surgical site, 3) Maximal ureteral dissection facilitates reimplantation, 4) Frozen section from the stricture is mandatory to rule out malignancy.

**CONCLUSIONS:**

In our experience, UAS repair is feasible and reproducible using a minimally invasive robotic approach. Comparative studies with open surgical approach are warranted.

## ARTICLE INFO

**Available at: https://www.intbrazjurol.com.br/video-section/20180794_Garisto_et_al**

**Int Braz J Urol. 2019; 45 (Video #25): 1275-6**

